# Factors influencing the likelihood of dental service checkup: results from a survey in Saudi Arabia

**DOI:** 10.3389/froh.2023.1208929

**Published:** 2023-12-15

**Authors:** Majed Almutairi, Gerry McKenna, Ibrahim Alsumaih, Rasha Alhazzaa, Ciaran O’Neill

**Affiliations:** ^1^ Centre for Public Health, School of Medicine, Dentistry and Biomedical Sciences, Queen’s University Belfast, Belfast, United Kingdom; ^2^ Public Health Department, School of Health Sciences, Saudi Electronic University, Riyadh, Saudi Arabia; ^3^ Department of Medical Support Services, King Fahad Hofuf Hospital, Ministry of Health, Hofuf, Saudi Arabia; ^4^ Health Informatics Department, School of Health Sciences, Saudi Electronic University, Riyadh, Saudi Arabia

**Keywords:** Saudi Arabia, dental service use, regional variation, dental checkup, income

## Abstract

**Background:**

The funding and delivery of healthcare including dental care in the Kingdom of Saudi Arabia (KSA, or Saudi Arabia) is undergoing a process of reform. To inform this process, it is important that policymakers are aware of the relationships between service use, specific types of use, and the factors that influence this. Currently, there is a paucity of research in this area in KSA that examines dental service use for checkups at a national level and none that examines differences in this use across regions or that examines explicitly the role of income.

**Aims:**

This study uses the most recent version of the Saudi Health Interview Survey (SHIS) to examine the relationships between the use of dental services for a checkup and socio-demographic characteristics of respondents. Particular focus is given to the differences between regions in service use and the role of socio-demographics within regions.

**Methods:**

Data were taken from SHIS 2013. Descriptive statistics (means and standard errors) were used to characterize the sample. Logistic regression analyses were used to examine the relationship between checkups in the past 12 months and a range of covariates including income and region. The analysis was repeated for sub-samples based on specific regions. No attempt was made to impute missing values.

**Results:**

A sample of 7603 respondents provided complete data for analysis. Fifty-one per cent of the respondents were male, 29% were educated at least to degree level, 25% reported that they floss at least once per day, 69% reported that they brushed their teeth at least once per day, and 11% reported that they had visited the dentist for a checkup in the preceding 12 months. Logistic regression analyses revealed income, region, and oral hygiene habits to be among the significant determinants of the likelihood of dental checkup in the preceding 12 months. In logistic regression analyses at the regional level, different relationships were evident between checkups and socio-demographic characteristics across regions.

**Conclusion:**

Region and income are significant determinants of dental service use for checkups. Differences exist between regions in the relationship between socio-demographic characteristics and the likelihood of getting checkups. Policy changes should reflect the potential differences they might have across regions for which the role of socio-demographic characteristics varies.

## Introduction

All healthcare systems face the common challenge of resource scarcity. In recent years, for many systems these challenges have been exacerbated by a combination of factors that have affected demand and supply. With respect to demand, factors such as population aging (1, 2) and lifestyle choices related to diet (3, 4) and exercise (5, 6) have helped fuel an increase in non-communicable disease, multi-morbidity, and expenditures on healthcare associated with meeting that demand. With respect to supply, the seeming paradox of rapid technological advance coupled with slow productivity growth has also served to produce spiraling healthcare expenditures, and among those systems wherein access to care relies heavily on public funding, pressures on public funds (7–9). These pressures extend to oral healthcare, where many countries also face challenges of how to meet the needs of their populations for dental care (10). These challenges include finance issues, shortage of manpower, and issues of equity of access (11).

Dental health services play an important role in oral health, which has in turn been shown to affect individual general health, quality of life, and self-esteem (12–14). Regular dental checkups play a role in care. For instance, according to Petersen (2003), most tooth loss occurs due to the progression of diseases mainly because people do not make use of dental services (15). It is also worth noting that major oral disorders are among the 35 causes of disability adjusted life years (DALYs) as indicated by Vos et al.’s (2012) research findings (16). This highlights the importance of dental checkups, in preventing and managing oral diseases, which ultimately leads to better global health outcomes. In the Kingdom of Saudi Arabia (KSA, or Saudi Arabia), the provision of healthcare is a government priority (17). As the government has sought to diversify the economy and introduce structural reforms under Saudi Arabia's Vision 2030 initiatives, so the need to consider how best to fund and deliver services has grown (18). The reforms have included efforts to increase tax and reduce government subsidization of public services—generally as well as by the promotion of privately provided and funded healthcare (19). This has seen significant changes in healthcare in recent years (17).. Access to care is seen as a fundamental right—the constitution of Saudi Arabia stating that the government is responsible for providing healthcare, and that every citizen and resident has a right to access these services (20, 21). This had led to the government of the KSA paying for all basic dental care (22). Health services are provided to the people by public (governmental) and separately private health sectors. The government also allocates funds annually to other government sectors, including the Ministry of Interior, the Ministry of National Guard, the Ministry of Defence, and the Ministry of Education, who operate health insurance programs and facilities for their employees and families, and to the public in general in, for example, the event of an emergency (20, 23). However, it is unlikely that this approach will persist.

A number of studies have examined the use of dental services as well as oral hygiene habits/self-care in Saudi Arabia using both national and regional specific datasets (24–28). According to El Bcheraoui et al., 1.5 million individuals (that is 11.5% of the population) in Saudi Arabia visited a dental clinic for routine checkup in 2013, 71.5% reported brushing their teeth at least once a day. Up to 30.3% used Miswak—akin to a toothpick—at least once a day (24). Studies on the use of dental services in Saudi Arabia have produced conflicting results with respect to the role of socio-economic status. Some have shown utilization of dental services to exhibit a pro-rich socio-economic gradient with respect to income and/or education, though other studies found no relationship between education or income and dental service use in Saudi Arabia (24–26, 28, 29).

Location can play an important role in access to care and, as a result, in relation to utilization of care (30, 31). Whether as a result of where dentists choose to practice or where government chooses to provide services, both of which may see services concentrated in population centers, travel costs may add to the costs of consuming dental care borne by the user/parent and as a result deter utilization of the service for those who, for example, reside away from urban centers. Consistent with this, studies in Saudi Arabia have shown that utilization of oral services was lower in rural areas than in urban areas (27). Similarly, various socio-demographic factors may contribute to differences in either costs or perceived benefits from use of services and underpin differences in use. For example, those who are more affluent may be better able to afford dental care—where their exist co-pays—and/or may attach a higher value to oral health in monetary terms than those who are less well-off and as a result be expected to be more likely to visit the dentist (32). This will similarly apply to education—likely to be positively correlated with income—in that those who are better educated may have higher degrees of health literacy and be better able to identify needs, including that for preventive care, and be expected to make greater use of the services as a result (32, 33). Other studies in Saudi Arabia have also shown income and education to be related to oral health and to use of dental services (34). The number of patients who use dental services was higher among patients whose income was higher in Abha, a city in Saudi Arabia (25). A study conducted in the city of Jazan in Saudi Arabia similarly shows that individuals who had completed only their primary education were half as likely to having regular dental appointments compared with those who had completed higher levels of education (26).

Other studies have shown differences in use related to gender that may be grounded in the value attached to the aesthetic benefits of good oral health or simply in the commonly observed greater likelihood of women to use medical services than men (35). These findings have been echoed in other studies in Saudi Arabia, with men exhibiting a lower likelihood of visiting the dentist when adjusted for covariates than women (28). Other factors that may influence use include marital status (through, e.g., pester power—the spouse pestering their partner to use the services) and age as a result of increasing oral health needs. Studies in Saudi Arabia have demonstrated the role of marital status, one study in the city of Abha in Saudi Arabia finding that participants who were single were more likely to not use dental services than participants who were married (25). In addition, a lack of perceived dental needs was found to be the most significant barrier, which prevented 70.2% of the people from getting dental treatment in Jazan, Saudi Arabia (26).

While studies within Saudi Arabia have examined the role of socio-demographic characteristics in service use, they have not hitherto examined differences in use between regions and only one study has used national data to examine the role of income (36). The study by El Bcheraoui et al. (24), which also used national data to examine use of services for checkups, did not look at the role of income or region where potentially distinct issues in access or cultural norms may give rise to different patterns of service use for checkups (11, 24). The purpose of this analysis was to add to our body of knowledge by looking at the role of income, region, and income within regions (the potential for its role to differ across regions) in utilization of dental services for checkups. In addition to examining the role of socio-demographic status at a national level, the study seeks to determine if the roles of socio-demographic variables and socio-economic status in particular differ between regions, providing insights into patterns of service use for checkups of potential value to policymakers. While the study by El Bcheraoui et al. also contains details on use of services for “dental complaints” as this was a rather broad term, covering the gamut of restorative services, orthodontics, and periodontics, we focused attention on checkups to give sharper focus to the examination.

## Materials

The Saudi Health Information Survey (SHIS) is a national multistage survey of individuals aged over 15 years old. The survey used in this study was conducted on behalf of the Ministry of Health across 13 administrative regions in Saudi Arabia in 2013. These regions are Al Riyadh; AlQassim; Makkah Al Moukarrama; Tabuk; Hail; Al-Jouf; Al-Baha; Eastern Region; Northern Borders; Madinah; Jazan; Aseer; and Najran. To recruit study participants and to ensure that the survey findings were representative of the population of Saudi Arabia, survey respondents were recruited using a multistage stratified probability sampling method. The stratification was based on the Kingdom's 13 regions. Approximately 12,000 households were randomly selected for participation in the survey from across the 13 regions. A total of 10,827 individuals completed the survey and were contacted by local primary care centers to participate in this study. The primary sampling units are tiny clusters of households that have been broken up and designated by the Census Bureau of the Kingdom of Saudi Arabia. On average, there are approximately 140 households in each of these clusters. The survey gathers information on a wide variety of socio-demographic factors, including age, gender, educational achievement, marital status, and income. The questionnaire also provides data on life style factors such as diet, level of physical activities, and utilization of healthcare services (24).

## Methods

Descriptive statistics—mean, standard errors, and 95% confidence intervals—across the sample and for sub-samples based on specific regions were used to describe the data and compare differences across regions. Multivariable logistic regression analyses were used to examine the relationship between use of dental services for checkups and respondent characteristics including age, gender (male vs. female), marital status and oral hygiene habits. Service use was dichotomized to capture use in the past 12 months (=1) vs. non-use in this time period (=0). (While the survey identified once and more than once as options, we redefined it as having visited or not, for ease of exposition.) Income was entered into the analysis as a series of categories matching the eight groups contained in the survey—less than 3,000 riyals per month, 3,000–5,000 riyals per month, 5,000 Riyals to less than 7,000 Riyals per month, 7,000 Riyals to less than 10,000 Riyals per month, 10,000 Riyals to less than 15,000 Riyals per month, 15,000 Riyals to less than 20,000 Riyals per month, 20,000 Riyals to less than 30,000 Riyals per month, and 30,000 Riyals or more per month. The corresponding values in Euro are reported in Supplementary Appendix S2. The lowest income group provided the base category against which each of the others was compared. Region was also entered as a series of categorical variables matching the 13 in the survey with Riyadh providing the base category against which the others were compared. Precise details of the survey wording are available from the SHIS results (37). The analysis was confined to those respondents who provided complete data; no attempt was made to impute data. As attention was given to variations among individuals rather than attempting to provide national estimates, no attempt was made to weight the data.

To allow for the possibility that distinct relationships may exist between the likelihood of dental checkup use and respondent characteristics across regions, logistic regression analyses were rerun for sub-groups based on individual regions. Separate analyses for each region were undertaken rather than using models in which interaction terms between, for example, region and individual characteristics were used to allow for the possibility of distinct relationships between covariates and checkup use across regions. A hierarchical logistic regression was also undertaken to evidence the value-added from the inclusion of income and region among the explanatory variables. In the interest of brevity, not all regional analyses are reported, rather those for which the existence of significant differences between regions were evident are reported. Odds ratios were compared across regions based on the point estimate and the associated 95% confidence interval.

Variables were selected for use in the regression analysis based on the Andersen model (38). The model (39) seeks to understand checkup use in terms of factors that can be grouped under headings of need, predisposing, and enabling (40). Predisposing factors, for example, may be related to age or education where perceived risk of tooth decay, for example, may vary across groups for whom the risk of tooth decay varies due to the accumulation of restorative care over time (13) or between whom degrees of health literacy and perceived risk of dental problems varies with education (13). Similarly, with respect to enabling factors, aspects such as income were included, which may affect the affordability of the service or the ability to travel to consume it, and thus the likelihood of use (41). With respect to needs such as arising from the experience of dental pain, unsurprisingly these were not captured directly in a survey of this type though oral hygiene habits—such as use of brushing and flossing that are captured in the survey—may offer an indirect measure of them. While some care is warranted in the grouping of variables under particular headings—for example, oral hygiene could equally be interpreted as a predisposing factor—in as much as they help identify variables that may explain variations in use, the approach has been shown to be helpful (39).

All analyses were conducted using the software Stata Version 16.0.

## Results

A total of 7,603 usable responses—with complete data—were obtained from the survey. Table 1 presents the descriptive statistics for this sample. As shown, 11% had visited the dentist for a checkup in the preceding 12 months. 51.5% were male, 18% of the respondents were smokers, and 29.2% were educated to degree level or above. With respect to age, those between 25 and 34 represent the largest age group in the sample, with 27.4%. Approximately 67% of the participants were married. Participants who reported that they floss their teeth at least once a day were 25% of the sample, while 69% reported that they brush their teeth at least once a day. Roughly 3.5% of the sample reported that they use Miswak (tooth stick) at least once a day. The lowest income group comprised 16.5% of the sample, while the highest income group represented 2.4% of the sample. Across the 13 regions in Saudi Arabia, participants from Riyadh region were the largest group, with 15.96% of the sample.

**Table 1:** Descriptive statistics of the usable sample.

Mean estimation	Number of obs = 7,603			
	Mean	Std. err.	[95% conf. interval]	
Oral checkup	0.1136	0.0036	0.1065	0.1208
Male	0.5152	0.0057	0.5040	0.5264
Education				
Illiterate	0.2093	0.0047	0.2001	0.2184
High school	0.4980	0.0057	0.4867	0.5092
University and above	0.2928	0.0052	0.2825	0.3030
Smoker	0.1839	0.0044	0.1752	0.1926
Age				
Age 15–24	0.1991	0.0046	0.1902	0.2081
Age 25–34	0.2744	0.0051	0.2643	0.2844
Age 35–44	0.2304	0.0048	0.2210	0.2399
Age 45–54	0.1367	0.0039	0.1289	0.1444
Age 55–64	0.0759	0.0030	0.0699	0.0818
Age +65	0.0835	0.0032	0.0773	0.0897
Marital status				
Married	0.6704	0.0054	0.6598	0.6810
Not married	0.2442	0.0049	0.2346	0.2539
Divorced, separated, and widowed	0.0854	0.0032	0.0791	0.0916
Oral habits				
Floss				
Do not floss	0.8393	0.0042	0.8310	0.8475
Less than once per day	0.0906	0.0033	0.0842	0.0971
Once per day	0.0481	0.0025	0.0433	0.0530
2+ times per day	0.0220	0.0017	0.0187	0.0253
Miswak				
Never	0.4890	0.0057	0.4778	0.5003
Less than once a day	0.1651	0.0043	0.1567	0.1734
Once a day	0.1586	0.0042	0.1504	0.1668
Twice a day, or more	0.1873	0.0045	0.1785	0.1961
Brush				
Never	0.1920	0.0045	0.1832	0.2009
Less than once a day	0.1161	0.0037	0.1089	0.1233
Once a day	0.3393	0.0054	0.3287	0.3500
Twice a day or more	0.3525	0.0055	0.3418	0.3632
Income (Saudi Riyals)				
Less than 3,000	0.1655	0.0043	0.1571	0.1738
3,000–5,000	0.1711	0.0043	0.1626	0.1796
5,000–7,000	0.1635	0.0042	0.1552	0.1718
7,000–10,000	0.1947	0.0045	0.1858	0.2036
10,000–15,000	0.1695	0.0043	0.1611	0.1780
15,000–20,000	0.0798	0.0031	0.0737	0.0859
20,000–30,000	0.0312	0.0020	0.0273	0.0351
More than 30,000	0.0247	0.0018	0.0212	0.0282
Riyadh	0.1597	0.0042	0.1514	0.1679
Jouf	0.0408	0.0023	0.0363	0.0452
Western	0.1576	0.0042	0.1494	0.1658
Almadina	0.0622	0.0028	0.0568	0.0676
Qassem	0.0320	0.0020	0.0280	0.0359
East	0.0702	0.0029	0.0645	0.0760
Aseer	0.0868	0.0032	0.0805	0.0931
Tabouk	0.0533	0.0026	0.0482	0.0583
Haiel	0.0642	0.0028	0.0587	0.0697
Northern	0.0537	0.0026	0.0486	0.0587
Jazan	0.0810	0.0031	0.0749	0.0872
Najran	0.0710	0.0029	0.0652	0.0768
Baha	0.0676	0.0028	0.0619	0.0732

In Table 2, the results of a logistic regression show that there are differences in the likelihood of a visit for a checkup inter alia by region and income. People in the higher-income groups were more likely to have visited the dentist for a checkup in the last 12 months compared with those in the lowest-income group. Similarly, people who were better educated were more likely to have visited a dentist for a checkup than those who were less well educated. Participants aged 15–24 were more likely to have visited a dentist for a checkup in the last 12 months than other age groups. Notably differences in the likelihood of a checkup visit were evident across regions. In Tables 3a–c, moreover, it is seen that differences in the role of socio-demographic variables also exist between regions. While income is a significant determinant of the likelihood of a checkup in Riyadh and Jazan, for example, this is not the case in Baha; similarly, while age is significant in both the former regions, this is not the case in Baha. In Table 4, the results of the hierarchical model are reported. As can be seen from the likelihood ratio results and from the Akaike information criterion (AIC) statistics, the inclusion of income and region add to the explanatory power of the model.

**Table 2:** Logistic regression of oral checkup for the entire sample.

Logistic regression		Number of obs = 7,603				
		Wald *χ*^2^ (39) = 387.63				
		Prob > *χ*^2^ = 0.0000				
Log pseudolikelihood = −2,453.3583		Pseudo *R*^2^ = 0.0886				
		Robust				
Oral checkup	Odds ratio	Std. err.	z	*P* > z	[95% conf. interval]	
Male [ref. female]	0.9525	0.0885	−0.5200	0.6000	0.7938	1.1428
Education [ref. illiterate]						
High school	1.5063	0.2361	2.6100	0.0090	1.1078	2.0480
University and above	1.8622	0.3065	3.7800	0.0000	1.3487	2.5712
Smoker [ref. non-smoker]	1.0510	0.1126	0.4600	0.6420	0.8520	1.2965
Age [ref. 65 and over]						
Age 15–24	0.5745	0.1346	−2.3600	0.0180	0.3629	0.9095
Age 25–34	0.5406	0.1106	−3.0100	0.0030	0.3620	0.8072
Age 35–44	0.5304	0.1068	−3.1500	0.0020	0.3574	0.7870
Age 45–54	0.5055	0.1038	−3.3200	0.0010	0.3381	0.7559
Age 55–64	0.5605	0.1286	−2.5200	0.0120	0.3575	0.8789
Marital status [ref. married]						
Not married	1.0992	0.1407	0.7400	0.4600	0.8552	1.4127
Divorced, separated, or widowed	0.8055	0.1386	−1.2600	0.2090	0.5749	1.1285
Oral habits						
Floss [ref. do not floss]						
Less than once per day	1.8055	0.1977	5.4000	0.0000	1.4568	2.2378
Once per day	1.8949	0.2695	4.4900	0.0000	1.4340	2.5040
2+ times per day	2.1040	0.4368	3.5800	0.0000	1.4007	3.1604
Miswak [ref. Never]						
Less than once a day	1.0453	0.1208	0.3800	0.7020	0.8333	1.3111
Once a day	1.5110	0.1603	3.8900	0.0000	1.2272	1.8603
Twice a day, or more	1.4222	0.1581	3.1700	0.0020	1.1438	1.7684
Brush [ref. Never]						
Less than once a day	1.4418	0.3039	1.7400	0.0830	0.9538	2.1793
Once a day	2.5055	0.4085	5.6300	0.0000	1.8202	3.4489
Twice a day or more	3.9620	0.6525	8.3600	0.0000	2.8690	5.4714
Income (Saudi Riyals) [ref. Less than 3,000]						
3,000–5,000	1.0241	0.1612	0.1500	0.8800	0.7522	1.3941
5,000–7,000	1.3447	0.2086	1.9100	0.0560	0.9922	1.8225
7,000–10,000	1.4547	0.2204	2.4700	0.0130	1.0810	1.9575
10,000–15,000	1.2330	0.1963	1.3200	0.1880	0.9024	1.6846
15,000–20,000	1.5029	0.2641	2.3200	0.0200	1.0650	2.1207
20,000–30,000	1.6226	0.3537	2.2200	0.0260	1.0584	2.4876
More than 30,000	1.1667	0.3271	0.5500	0.5820	0.6735	2.0213
Regions [ref. Baha]						
Riyadh	2.4929	0.5735	3.9700	0.0000	1.5881	3.9131
Jouf	0.8705	0.3373	−0.3600	0.7200	0.4073	1.8605
Western	2.9946	0.6855	4.7900	0.0000	1.9120	4.6902
Almadina	2.1172	0.5682	2.8000	0.0050	1.2512	3.5825
Qassem	2.4721	0.7421	3.0200	0.0030	1.3726	4.4523
East	2.8456	0.7078	4.2000	0.0000	1.7477	4.6333
Aseer	0.9997	0.2770	0.0000	0.9990	0.5809	1.7207
Tabouk	2.9282	0.7696	4.0900	0.0000	1.7494	4.9013
Haiel	2.0103	0.5485	2.5600	0.0100	1.1777	3.4316
Northern	1.7632	0.5175	1.9300	0.0530	0.9919	3.1342
Jazan	3.4654	0.8372	5.1400	0.0000	2.1583	5.5642
Najran	2.3061	0.6098	3.1600	0.0020	1.3734	3.8721
_cons	0.0151	0.0044	−14.2900	0.0000	0.0085	0.0268

**Table 3a:** Logistic regression of regions Riyadh.

Logistic regression		Number of obs = 1,184				
		Wald *χ*^2^ (26) = 86.00				
		Prob > *χ*^2^ = 0.0000				
Log pseudolikelihood = −427.12669		Pseudo *R*^2^ = 0.1166				
Oral checkup	Odds ratio	Robust				
		Std. err.	z	*P* > z	[95% conf. interval]	
Male [ref. female]	0.7586	0.1677	−1.2500	0.2110	0.4919	1.1699
Education [ref. illiterate]						
High school	1.1713	0.4210	0.4400	0.6600	0.5791	2.3695
University and above	1.8865	0.7001	1.7100	0.0870	0.9115	3.9045
Smoker [ref. non-smoker]	1.2107	0.3195	0.7200	0.4690	0.7218	2.0309
Age [ref. 65 and over]						
Age 15–24	0.4295	0.2456	−1.4800	0.1390	0.1400	1.3175
Age 25–34	0.2934	0.1506	−2.3900	0.0170	0.1073	0.8024
Age 35–44	0.2046	0.1027	−3.1600	0.0020	0.0765	0.5474
Age 45–54	0.4177	0.2054	−1.7800	0.0760	0.1593	1.0951
Age 55–64	0.3090	0.1799	−2.0200	0.0440	0.0987	0.9673
Marital status [ref. married]						
Not married	0.9738	0.2869	−0.0900	0.9280	0.5467	1.7348
Divorced, separated, or widowed	0.9101	0.3067	−0.2800	0.7800	0.4702	1.7616
Oral habits						
Floss [ref. do not floss]						
Less than once per day	1.8621	0.4209	2.7500	0.0060	1.1956	2.9002
Once per day	2.4645	0.7908	2.8100	0.0050	1.3140	4.6222
2+ times per day	2.3801	1.2041	1.7100	0.0870	0.8830	6.4154
Miswak [ref. Never]						
Less than once a day	1.1743	0.3465	0.5400	0.5860	0.6586	2.0938
Once a day	1.3062	0.3152	1.1100	0.2680	0.8139	2.0962
Twice a day, or more	1.5798	0.3831	1.8900	0.0590	0.9822	2.5409
Brush [ref. Never]						
Less than once a day	10.1429	11.0759	2.1200	0.0340	1.1931	86.2307
Once a day	17.8137	18.4637	2.7800	0.0050	2.3361	135.8361
Twice a day or more	31.1430	32.4291	3.3000	0.0010	4.0458	239.7255
Income (Saudi Riyals) [ref. Less than 3,000 Riyals]						
3,000–5,000	1.1982	0.6804	0.3200	0.7500	0.3937	3.6465
5,000–7,000	2.0912	1.1373	1.3600	0.1750	0.7202	6.0717
7,000–10,000	3.2625	1.7088	2.2600	0.0240	1.1687	9.1072
10,000–15,000	2.1659	1.1502	1.4600	0.1460	0.7649	6.1331
15,000–20,000	2.1609	1.1897	1.4000	0.1620	0.7345	6.3570
20,000–30,000	1.8648	1.1494	1.0100	0.3120	0.5572	6.2414
More than 30,000	1.0000	(empty)				
_cons	0.0068	0.0077	−4.3900	0.0000	0.0007	0.0629

**Table 3b:** Logistic regression of Jazan.

Logistic regression		Number of obs = 613				
		Wald *χ*^2^ (25) = 90.22				
		Prob > *χ*^2^ = 0.0000				
Log pseudolikelihood = −194.88657		Pseudo *R*^2^ = 0.2431				
Oral checkup	Odds ratio	Robust				
		Std. err.	z	*P* > z	[95% conf. interval]	
Male [ref. female]	0.8985	0.2984	−0.3200	0.7470	0.4685	1.7228
Education [ref. illiterate]						
High school	0.5451	0.2609	−1.2700	0.2050	0.2133	1.3929
University and above	1.1069	0.5494	0.2000	0.8380	0.4184	2.9283
Smoker [ref. non-smoker]	1.2180	0.5303	0.4500	0.6510	0.5188	2.8594
Age [ref. 65 and over]						
Age 15–24	0.2227	0.1604	−2.0900	0.0370	0.0543	0.9133
Age 25–34	0.2257	0.1559	−2.1500	0.0310	0.0583	0.8741
Age 35–44	0.3789	0.2355	−1.5600	0.1180	0.1121	1.2811
Age 45–54	0.2728	0.1721	−2.0600	0.0400	0.0792	0.9395
Age 55–64	0.1744	0.1119	−2.7200	0.0060	0.0496	0.6131
Marital status [ref. married]						
Not married	1.4890	0.6269	0.9500	0.3440	0.6524	3.3985
Divorced, separated, or widowed	0.9885	0.5066	−0.0200	0.9820	0.3620	2.6989
Oral habits						
Floss [ref. do not floss]						
Less than once per day	3.4489	1.4058	3.0400	0.0020	1.5514	7.6673
Once per day	1.9790	1.4001	0.9600	0.3350	0.4946	7.9184
2+ times per day	1.0000	(empty)				
Miswak [ref. Never]						
Less than once a day	1.4496	0.6006	0.9000	0.3700	0.6435	3.2654
Once a day	6.2230	2.2234	5.1200	0.0000	3.0894	12.5349
Twice a day, or more	3.2125	1.2976	2.8900	0.0040	1.4556	7.0902
Brush [ref. Never]						
Less than once a day	3.7125	2.8391	1.7200	0.0860	0.8293	16.6194
Once a day	7.6690	4.1783	3.7400	0.0000	2.6362	22.3101
Twice a day or more	11.6624	6.5394	4.3800	0.0000	3.8860	35.0010
Income (Saudi Riyals) [ref. Less than 3,000 Riyals]						
3,000–5,000	0.8032	0.4317	−0.4100	0.6830	0.2801	2.3030
5,000–7,000	1.3295	0.6962	0.5400	0.5860	0.4764	3.7103
7,000–10,000	2.2961	1.0691	1.7900	0.0740	0.9218	5.7190
10,000–15,000	1.7899	0.8943	1.1700	0.2440	0.6723	4.7654
15,000–20,000	3.3466	1.7144	2.3600	0.0180	1.2262	9.1337
20,000–30,000	5.3908	3.3498	2.7100	0.0070	1.5948	18.2215
More than 30,000	1.0000	(empty)				
_cons	0.0234	0.0143	−6.1600	0.0000	0.0071	0.0773

**Table 3c:** Logistic regression of Baha.

Logistic regression		Number of obs = 429				
		Wald *χ*^2^ (23) = 54.10				
		Prob > *χ*^2^ = 0.0003				
Log pseudolikelihood = −72.409591		Pseudo *R*^2^ = 0.2173				
Oral checkup	Odds ratio	Robust				
		Std. err.	z	*P* > z	[95% conf. interval]	
Male [ref. female]	1.2554	0.7755	0.3700	0.7130	0.3741	4.2129
Education [ref. illiterate]						
High school	0.8435	1.7171	−0.0800	0.9330	0.0156	45.5889
University and above	11.3555	19.2971	1.4300	0.1530	0.4062	317.4687
Smoker [ref. non-smoker]	1.1867	0.7094	0.2900	0.7750	0.3677	3.8301
Age [ref. 65 and over]						
Age 15–24	4.4444	7.2057	0.9200	0.3580	0.1852	106.6307
Age 25–34	0.6069	0.8356	−0.3600	0.7170	0.0408	9.0183
Age 35–44	0.3801	0.4809	−0.7600	0.4450	0.0319	4.5363
Age 45–54	0.6541	1.0118	−0.2700	0.7840	0.0315	13.5616
Age 55–64	1.0000	(omitted)				
Marital status [ref. Married]						
Not married	0.3989	0.2016	−1.8200	0.0690	0.1481	1.0743
Divorced, separated, or widowed	0.6470	0.9083	−0.3100	0.7560	0.0413	10.1350
Oral habits						
Floss [ref. do not floss]						
Less than once per day	1.8298	1.3169	0.8400	0.4010	0.4465	7.4991
Once per day	1.0000	(empty)				
2+ times per day	1.0000	(empty)				
Miswak [ref. Never]						
Less than once a day	1.5673	0.9874	0.7100	0.4760	0.4559	5.3880
Once a day	0.5744	0.4711	−0.6800	0.4990	0.1151	2.8668
Twice a day, or more	1.4281	1.0768	0.4700	0.6370	0.3258	6.2602
Brush [ref. Never]						
Less than once a day	1.9291	2.8667	0.4400	0.6580	0.1048	35.5026
Once a day	1.8611	2.6923	0.4300	0.6680	0.1092	31.7056
Twice a day or more	4.3390	5.9971	1.0600	0.2880	0.2890	65.1436
Income [ref. Less than 3,000 Riyals]						
3,000–5,000	0.4536	0.5693	−0.6300	0.5290	0.0388	5.3079
5,000–7,000	2.1474	3.3564	0.4900	0.6250	0.1003	45.9571
7,000–10,000	2.6033	3.6317	0.6900	0.4930	0.1691	40.0837
10,000–15,000	1.3489	1.7962	0.2200	0.8220	0.0992	18.3410
15,000–20,000	2.3262	2.9227	0.6700	0.5020	0.1982	27.2984
20,000–30,000	1.9768	3.1581	0.4300	0.6700	0.0863	45.2724
More than 30,000	1.0000	(empty)				
_cons	0.0051	0.0047	−5.7100	0.0000	0.0008	0.0314

**Table 4:** Hierarchical logistic regression.

Block 1: Male, High school University and above, smoker, Age 15–24, Age 25–34, Age 35–44, Age 45–54, Age 55–64, Not married, Divorced, separated, or widowed, Less than once per day, Once per day, 2+ times per day, Twice a day, or more						
Logistic regression	Number of obs = 7,603					
	LR *χ*^2^ (20) = 370.51					
	Prob > *χ*^2^ = 0.0000					
Log likelihood = −2506.6407	Pseudo *R*^2^ = 0.0688					
Oral checkup	Odds ratio	Std. err.	Z	*P* > z	[95% conf. interval]	
Male [ref. female]	0.9757	0.0870	−0.2800	0.7830	0.8193	1.1621
Education [ref. illiterate]						
High school	1.7145	0.2585	3.5800	0.0000	1.2758	2.3040
University and above	2.1559	0.3325	4.9800	0.0000	1.5935	2.9167
Smoker [ref. non-smoker]	1.0925	0.1152	0.8400	0.4020	0.8885	1.3432
Age [ref. 65 and over]						
Age 15–24	0.5645	0.1267	−2.5500	0.0110	0.3636	0.8763
Age 25–34	0.5456	0.1062	−3.1100	0.0020	0.3725	0.7991
Age 35–44	0.5233	0.1012	−3.3500	0.0010	0.3583	0.7643
Age 45–54	0.5239	0.1041	−3.2500	0.0010	0.3549	0.7734
Age 55–64	0.5746	0.1286	−2.4800	0.0130	0.3706	0.8908
Marital status [ref. married]						
Not married	1.0754	0.1301	0.6000	0.5480	0.8484	1.3631
Divorced, separated, or widowed	0.7699	0.1321	−1.5200	0.1270	0.5501	1.0775
Floss [ref. do not floss]						
Less than once per day	1.9593	0.2114	6.2300	0.0000	1.5859	2.4206
Once per day	1.9753	0.2734	4.9200	0.0000	1.5059	2.5908
2+ times per day	2.0482	0.4073	3.6100	0.0000	1.3871	3.0245
Miswak [ref. Never]						
Less than once a day	1.0295	0.1166	0.2600	0.7980	0.8246	1.2853
Once a day	1.4833	0.1534	3.8100	0.0000	1.2112	1.8166
Twice a day, or more	1.3894	0.1473	3.1000	0.0020	1.1288	1.7102
Brush [ref. Never]						
Less than once a day	1.4285	0.2942	1.7300	0.0830	0.9541	2.1389
Once a day	2.7752	0.4433	6.3900	0.0000	2.0291	3.7955
Twice a day or more	4.5623	0.7252	9.5500	0.0000	3.3411	6.2298
_cons	0.0347	0.0071	−16.5200	0.0000	0.0233	0.0517

Block 2: 3,000–5,000, 5,000–7,000, 7,000–10,000,10,000–15,000,15,000–20,000, 20,000–30,000, More than 30,000						
Logistic regression		Number of obs = 7,603				
		LR *χ*^2^ (27) = 387.85				
		Prob > *χ*^2^ = 0.0000				
Log likelihood = −2,497.9749		Pseudo *R*^2^ = 0.0720				
Oral checkup	Odds ratio	Std. err.	z	*P* > z	[95% conf. interval]	
Male [ref. female]	0.9643	0.0865	−0.4100	0.6850	0.8088	1.1496
Education [ref. illiterate]						
High school	1.5436	0.2378	2.8200	0.0050	1.1413	2.0877
University and above	1.8459	0.2986	3.7900	0.0000	1.3443	2.5346
Smoker [ref. non-smoker]	1.0790	0.1142	0.7200	0.4720	0.8770	1.3277
Age [ref. 65 and over]						
Age 15–24	0.5616	0.1266	−2.5600	0.0100	0.3611	0.8736
Age 25–34	0.5438	0.1065	−3.1100	0.0020	0.3705	0.7981
Age 35–44	0.5168	0.1001	−3.4100	0.0010	0.3535	0.7555
Age 45–54	0.5036	0.1006	−3.4300	0.0010	0.3404	0.7448
Age 55–64	0.5580	0.1253	−2.6000	0.0090	0.3594	0.8665
Marital status [ref. married]						
Not married	1.1274	0.1387	0.9700	0.3300	0.8859	1.4347
Divorced, separated, or widowed	0.8107	0.1403	−1.2100	0.2250	0.5775	1.1381
Floss [ref. do not floss]						
Less than once per day	1.9273	0.2088	6.0600	0.0000	1.5586	2.3832
Once per day	1.9353	0.2689	4.7500	0.0000	1.4739	2.5412
2+ times per day	1.9868	0.3965	3.4400	0.0010	1.3436	2.9380
Miswak [ref. Never]						
Less than once a day	1.0304	0.1168	0.2600	0.7920	0.8251	1.2867
Once a day	1.4950	0.1551	3.8800	0.0000	1.2199	1.8322
Twice a day, or more	1.4093	0.1500	3.2200	0.0010	1.1439	1.7362
Brush [ref. Never]						
Less than once a day	1.3868	0.2859	1.5900	0.1130	0.9258	2.0774
Once a day	2.7091	0.4331	6.2300	0.0000	1.9804	3.7061
Twice a day or more	4.4168	0.7037	9.3200	0.0000	3.2322	6.0356
Income (Saudi Riyals) [ref. less than 3,000]						
3,000–5,000	1.0103	0.1581	0.0700	0.9480	0.7434	1.3730
5,000–7,000	1.3435	0.2051	1.9300	0.0530	0.9961	1.8120
7,000–10,000	1.4456	0.2135	2.5000	0.0130	1.0823	1.9310
10,000–15,000	1.2320	0.1915	1.3400	0.1800	0.9084	1.6708
15,000–20,000	1.5270	0.2640	2.4500	0.0140	1.0881	2.1430
20,000–30,000	1.7203	0.3723	2.5100	0.0120	1.1256	2.6291
More than 30,000	0.9820	0.2678	−0.0700	0.9470	0.5755	1.6758
_cons	0.0317	0.0069	−15.8700	0.0000	0.0207	0.0485

Block 3: Riyadh, Jouf, Western, Almadina, Qassem, East, Aseer, Tabouk, Haiel, Northern, Jazan, Najran						
Logistic regression		Number of obs = 7,603				
		LR *χ*^2^ (39) = 477.08				
		Prob > *χ*^2^ = 0.0000				
Log likelihood = −2,453.3583		Pseudo *R*^2^ = 0.0886				
Oral checkup	Odds ratio	Std. err.	z	*P* > z	[95% conf. interval]	
Male [ref. female]	0.9525	0.0865	−0.5400	0.5920	0.7971	1.1380
Education [ref. illiterate]						
High school	1.5063	0.2332	2.6500	0.0080	1.1121	2.0402
University and above	1.8622	0.3053	3.7900	0.0000	1.3505	2.5678
Smoker [ref. non-smoker]	1.0510	0.1120	0.4700	0.6410	0.8530	1.2950
Age [ref. 65 and over]						
Age 15–24	0.5745	0.1299	−2.4500	0.0140	0.3688	0.8949
Age 25–34	0.5406	0.1068	−3.1100	0.0020	0.3671	0.7961
Age 35–44	0.5304	0.1037	−3.2400	0.0010	0.3615	0.7782
Age 45–54	0.5055	0.1020	−3.3800	0.0010	0.3404	0.7508
Age 55–64	0.5605	0.1267	−2.5600	0.0100	0.3599	0.8729
Marital status [ref. married]						
Not married	1.0992	0.1354	0.7700	0.4430	0.8634	1.3993
Divorced, separated, or widowed	0.8055	0.1405	−1.2400	0.2150	0.5722	1.1338
Floss [ref. do not floss]						
Less than once per day	1.8055	0.1996	5.3400	0.0000	1.4538	2.2423
Once per day	1.8949	0.2669	4.5400	0.0000	1.4377	2.4974
2+ times per day	2.1040	0.4282	3.6500	0.0000	1.4119	3.1355
Miswak [ref. Never]						
Less than once a day	1.0453	0.1201	0.3900	0.7000	0.8344	1.3094
Once a day	1.5110	0.1608	3.8800	0.0000	1.2265	1.8614
Twice a day, or more	1.4222	0.1553	3.2200	0.0010	1.1481	1.7617
Brush [ref. Never]						
Less than once a day	1.4418	0.2994	1.7600	0.0780	0.9596	2.1661
Once a day	2.5055	0.4050	5.6800	0.0000	1.8251	3.4396
Twice a day or more	3.9620	0.6431	8.4800	0.0000	2.8823	5.4461
Income (Saudi Riyals) [ref. less than 3,000]						
3,000–5,000	1.0241	0.1622	0.1500	0.8810	0.7508	1.3968
5,000–7,000	1.3447	0.2084	1.9100	0.0560	0.9924	1.8221
7,000–10,000	1.4547	0.2187	2.4900	0.0130	1.0835	1.9530
10,000–15,000	1.2330	0.1954	1.3200	0.1860	0.9037	1.6821
15,000–20,000	1.5029	0.2654	2.3100	0.0210	1.0631	2.1245
20,000–30,000	1.6226	0.3580	2.1900	0.0280	1.0530	2.5005
More than 30,000	1.1667	0.3265	0.5500	0.5820	0.6742	2.0191
Region [ref. Baha]						
Riyadh	2.4929	0.5719	3.9800	0.0000	1.5901	3.9083
Jouf	0.8705	0.3392	−0.3600	0.7220	0.4056	1.8684
Western	2.9946	0.6815	4.8200	0.0000	1.9171	4.6779
Almadina	2.1172	0.5548	2.8600	0.0040	1.2668	3.5383
Qassem	2.4721	0.7380	3.0300	0.0020	1.3771	4.4379
East	2.8456	0.7053	4.2200	0.0000	1.7506	4.6255
Aseer	0.9997	0.2759	0.0000	0.9990	0.5820	1.7172
Tabouk	2.9282	0.7643	4.1200	0.0000	1.7556	4.8839
Haiel	2.0103	0.5455	2.5700	0.0100	1.1811	3.4218
Northern	1.7632	0.5152	1.9400	0.0520	0.9945	3.1261
Jazan	3.4654	0.8431	5.1100	0.0000	2.1511	5.5828
Najran	2.3061	0.6082	3.1700	0.0020	1.3752	3.8671
_cons	0.0151	0.0045	−13.9600	0.0000	0.0084	0.0272
Block	LL	LR	df	Pr > LR	AIC	BIC
1	−2,506.641	370.51	20	0	5,055.281	5,200.944
2	−2,497.975	17.33	7	0.0154	5,051.95	5,246.166
3	−2,453.358	89.23	12	0	4,986.717	5,264.169

## Discussion

Saudi Arabia has embarked on a swath of reforms in an effort to reduce its dependence on oil revenues for public finance. The healthcare reforms under Vision 2030 in Saudi Arabia are transforming the healthcare landscape, including dental care (42). These reforms have the potential to provide long-lasting financial support, particularly for dental care. This strategy could greatly help in overcoming the obstacles that currently prevent people with lower incomes from accessing oral health services (43). The healthcare reforms in Saudi Arabia are working toward creating a future in which dental services are easily available, integrated into the healthcare system, and in line with a broader goal of total wellbeing. Large, nationally representative surveys can provide valuable insights to help guide these policy reforms by identifying those factors that influence utilization and that government may need to take cognizance of when devising policies to address particular barriers or facilitators to service use. This includes the role of income and region, where the role of region can extend beyond rurality (44). Small localized studies are limited in their ability to shed light on such issues, perhaps lacking statistical power or representation across a breadth of regions. Similarly, the opportunity to pool results from smaller local studies is limited by the heterogeneity in sampling methods, the covariates included, and even how utilization is framed in survey questions (39). Only larger national studies provide an opportunity to examine variation in service use generally, including that for checkups as examined here, using data collected in a consistent manner. Of the two previous studies that have examined dental service use in Saudi Arabia using national data (24, 36), one included income but did not examine region while the other examined neither the role of region nor income. A recent systematic review of the literature on inequalities in dental service has demonstrated that those with lower incomes are consistently less likely to use dental services (29). The findings of Sahab et al. (36) echo this for Saudi Arabia and underscore the value of its inclusion in studies of service use. Our own findings echo this with respect to checkups. Our results are broadly consistent with those of Sahab et al., which suggest the relationship with income is non-linear, increasing before falling.

The same review has highlighted differences between geographic areas differentiated by rurality—those from rural areas exhibiting lower utilization (29)—a finding at variance with that by Sahab et al. though echoed in studies undertaken subsequent to this review (45, 46). In this study, we clearly demonstrate in Tables 2 and 4 the importance of including region and income among the list of explanatory variables when examining the likelihood of getting a checkup, while Tables 3a–3c underscore the potential for the role of individual characteristics to vary between regions. This study provides evidence that points to the existence of differences between regions in the likelihood of a checkup—those in Jazan being more likely to visit a dentist while those in Jouf and Aseer were less likely to visit one than were those in Riyadh—and of distinct patterns within regions between the likelihood of a checkup and socio-demographic variables. Interestingly, this is seen not just with respect to income or age but also oral hygiene habits where, for example, in Riyadh these were positively related to the likelihood of a checkup (as was the case nationally), but in Baha there is no significant relationship. The existence of regional effects—differences between regions and in relationships within regions—suggests a one-size-fits-all model whether related to financial support for access or health education messages that may lack nuance. Rather differences between regions that may reflect differences in customs—for example, how westernized diets are or how sharp income inequalities are within regions—may require adjustments to policy. For example, in those areas where diet is an issue health education may be prioritized, while in those areas where access to dental checkups is an issue, increased supply may be prioritized. Interestingly perhaps, given the role of cultural norms around chaperoning of women, there were no differences in the likelihood of a checkup related to gender. That women do not seem to have experienced an additional barrier arising from this cultural norm is reassuring given the importance of checkups for prevention.

Clearly, the use of dental services for checkups in Saudi Arabia is influenced by the interplay of region and other individual level variables including income and age. Tables 3a–3c, highlight the complex connection between individual socio-economic variables and regions that may relate to the availability of oral healthcare services in different geographical areas—enabling factors in the Andersen framework. Urban areas typically provide a broader choice of dentists within close proximity of each other that may serve to encourage regular dental checkups (and perhaps induce demand from the public). Conversely, rural regions sometimes encounter challenges related to inadequate accessibility to dental facilities (36). Equally though, there could be differences in culture between regions that have a significant effect on patterns of use. Similarly, with respect to income, this could directly impact use of checkups as an enabling factor—how easy it is for a person, for example, to travel to the dentist—as well as indirectly, for example, in terms of the value to them of investments in preventive care—those with lower incomes typically facing financial barriers that lead to delayed or skipped checkups (47). This may help explain the role of these variables in our analysis, but further research on rurality, access, and income are warranted.

Unlike Sahab et al, this study was able to explore the role of oral health habits that those with good oral hygiene habits are more likely to use services for checkups is consistent with previous work by the authors (39) that highlighted the importance of differentiating between the types of need and types of care provided when examining utilization. While we were unable to examine the relationship between hygiene habits and use of dentists for specific treatment, it may well be the case that distinct relationships exist in this regard. The absence of income and region in previous analyses—which we have shown to be significant—demonstrates their importance (44) and the importance of their inclusion (36).

Comparing the sample that we used with that we did not use due to non-response in particular items, we can see that among those who provide complete responses, the percentage of men is higher, 52% of the respondents were male compared with 42% in the excluded sample (reported in Supplementary Appendix S1 Table S5), while the percentage of individuals with higher education is higher in the used sample than in the excluded sample (those with degrees were 29% in the used sample and 17% in the excluded sample). We can only speculate as to why they did not provide complete responses, although it is conceivable that women and those who are less well educated may encounter greater barriers in completing surveys of this type. While the usable sample differs in some respects from the full sample in terms of its representativeness, it is unlikely given its size and the numbers across distinct groups we were able to use that this had a material effect on results.

The study has a number of limitations. First, the data on which the analyses are based are over 10 years old. Given various factors including service provision, access, oral hygiene habits, and income may have changed in the intervening period, the relationships described may have altered somewhat since. That said, the study provides a snapshot of relationships at that time that will provide a useful comparator for future work it will hopefully encourage. Second, we were unable to look at different types of needs that may have prompted a checkup, for example, pain, routine behavior, and worry related to another oral health issue (39). Data on how frequently the respondent visited the dentist might provide further useful insights into patterns of use—in addition to how recently they visited, but this was not available. Third, the used sample of respondents was more likely to be better educated and male than the sample excluded due to non-response. This may have introduced an element of non-response bias, although large numbers of both genders and all education groups remained in the used sample. Fourth, the data are cross-sectional, which prevents us from looking at how patterns of use changed as individual circumstances changed. Our findings must therefore be interpreted as associations rather than causal. Further research could address these issues and examine how patterns of service use change in light of any reforms enacted in Saudi Arabia.

## Conclusion

National surveys can provide valuable insights into the associations between socio-demographic characteristics and utilization of dental services for policymakers. While previous studies in Saudi Arabia have examined the role of income and provided insights into differences between regions, only two studies have made use of large national datasets to examine these issues. Only one study has examined income, and neither has examined the potential for differences across regions. Our study echoes the previous national study with respect to income and identifies the existence of differences between regions in service use related to checkups as well as patterns of service use. That there exists evidence to suggest that across and within regions there are inequalities in uptake of checkups suggests policymakers should undertake further work to satisfy themselves that these are not grounded in differential access rather than the preferences of respondents, and if they are, adopt bespoke policies to address the barriers that exist.

## Data Availability

The original contributions presented in the study are included in the article/**Supplementary Material**. Further inquiries can be directed to the corresponding author.
