# Supplementary material for: Factors influencing the likelihood of dental service checkup: results from a survey in Saudi Arabia
Source: Front Oral Health. 2023 Dec 15;4:1208929. doi: 10.3389/froh.2023.1208929 (PMC10755011; doi:10.3389/froh.2023.1208929)
Supplement: Supplementary file 1 [file Table1.docx]

**Appendix 1**

**Table 5: Means for the excluded sample**

**Table 5-a**

| *Mean of Male* | | | | |
| --- | --- | --- | --- | --- |
|  | | | | |
| Mean estimation Number of obs = 3,218 | | | | |
|  | Mean | Std. Err. | [95% Conf. Interval] | |
| Male | .428527 | .0087249 | .4114201 | .445634 |

**Table 5-b**

| *Mean of Education* | | | | |
| --- | --- | --- | --- | --- |
| Mean estimation Number of obs = 1,971 | | | | |
|  | Mean | Std. Err. | [95% Conf. Interval] | |
| Education |  |  |  |  |
| Can read and write | .3054287 | .0103772 | .2850773 | .3257802 |
| High school | .5200406 | .0112561 | .4979655 | .5421157 |
| University and above | .1745307 | .0085517 | .1577593 | .1913021 |

**Table 5-c**

|  | | | | |
| --- | --- | --- | --- | --- |
| *Mean Smoker* | | | | |
| Mean estimation Number of obs = 3,185 | | | | |
|  | | | | |
|  | Mean | Std. Err. | [95% Conf. Interval] | |
| Smoker | .1359498 | .006074 | .1240405 | .147859 |

**Table 5-d**

| *Mean Income* | | | | |
| --- | --- | --- | --- | --- |
| Mean estimation Number of obs = 1,283 | | | | |
|  | Mean | Std. Err. | [95% Conf. Interval] | |
| Income (Saudi Riyals) |  |  |  | |
| Less than 3,000 | .2213562 | .011595 | .1986089 | .2441035 |
| 3,000 to 5,000 | .2642245 | .0123145 | .2400658 | .2883832 |
| 5,000 to 7,000 | .1644583 | .010353 | .1441475 | .1847691 |
| 7,000 to 10,000 | .1644583 | .010353 | .1441475 | .1847691 |
| 10,000 to 15,000 | .1060016 | .0085977 | .0891345 | .1228686 |
| 15,000 to 20,000 | .0436477 | .0057062 | .0324532 | .0548422 |
| 20,000 to 30,000 | .014809 | .0033735 | .0081909 | .0214272 |
| 30,000 or more | .0210444 | .0040087 | .01318 | .0289088 |

**Table 5-e**

| Mean Age | | | | |
| --- | --- | --- | --- | --- |
| Mean estimation Number of obs = 3,219 | | | | |
|  | Mean | Std. Err. | [95% Conf. Interval] | |
|  |  |  |  |  |
| Age 15-24 | .269649 | .007823 | .2543104 | .2849875 |
| Age 25-34 | .2084498 | .0071606 | .1944101 | .2224895 |
| Age 35-44 | .1823548 | .0068069 | .1690085 | .195701 |
| Age 45-54 | .1494253 | .0062846 | .1371031 | .1617474 |
| Age 55-64 | .0885368 | .0050077 | .0787182 | .0983554 |

**Table 5-f**

| Mean Marital status | | | | |
| --- | --- | --- | --- | --- |
| Mean estimation Number of obs = 3,181 | | | | |
|  | **Mean** | **Std. Err.** | **[95% Conf. Interval]** | |
| Marital status |  |  |  |  |
| Married | .6082993 | .0086561 | .5913272 | .6252714 |
| Not Married | .3109085 | .0082081 | .2948149 | .3270022 |
| Divorced, Separated and Widowed | .0807922 | .0048326 | .0713169 | .0902675 |

**Table 5-g**

| Mean Floss | | | | |
| --- | --- | --- | --- | --- |
| Mean estimation Number of obs = 3,065 | | | | |
|  | **Mean** | **Std. Err.** | **[95% Conf. Interval]** | |
| Floss | 1.190538 | .0102918 | 1.170359 | 1.210718 |

**Table 5-h**

| Mean Miswak | | | | |
| --- | --- | --- | --- | --- |
| Mean estimation Number of obs = 3,033 | | | | |
|  | **Mean** | **Std. Err.** | **[95% Conf. Interval]** | |
| Miswak | 2.021431 | .021858 | 1.978573 | 2.064289 |

**Table 5-i**

| Mean Brush | | | | |
| --- | --- | --- | --- | --- |
| Mean estimation Number of obs = 3,116 | | | | |
|  | **Mean** | **Std. Err.** | **[95% Conf. Interval]** | |
|  |  |  |  |  |
| Brush | 2.737484 | .0196485 | 2.698959 | 2.776009 |

**Table 6: Means for Riyadh, Jouf, Jazan and Baha regions**

**Table 6-a : Mean Riyadh**

|  | | | | |
| --- | --- | --- | --- | --- |
| Mean estimation Number of obs = 1,184 | | | | |
|  | Mean | Std. Err. | [95% Conf. Interval] | |
|  |  |  |  |  |
| Male | .5160473 | .0145296 | .4875406 | .544554 |
|  |  |  |  |  |
| Education |  |  |  |  |
| Can’t read or write | .1410473 | .0101199 | .1211924 | .1609022 |
| High School | .5067568 | .0145358 | .478238 | .5352755 |
| University and above | .3521959 | .0138874 | .3249492 | .3794427 |
|  |  |  |  |  |
| Smoker | .1655405 | .0108059 | .1443396 | .1867415 |
|  |  |  |  |  |
| Income (Saudi Riyals) |  |  |  |  |
| Less than 3,000 | .0701014 | .0074232 | .0555373 | .0846654 |
| 3,000 to 5,000 | .1528716 | .0104627 | .132344 | .1733992 |
| 5,000 to 7,000 | .1418919 | .0101451 | .1219874 | .1617963 |
| 7,000 to 10,000 | .2373311 | .0123695 | .2130624 | .2615997 |
| 10,000 to 15,000 | .2195946 | .0120359 | .1959805 | .2432087 |
| 15,000 to 20,000 | .1326014 | .0098603 | .1132557 | .151947 |
| 20,000 to 30,000 | .0456081 | .0060659 | .0337071 | .0575091 |
|  |  |  |  |  |
| Age 15-24 | .1537162 | .0104864 | .1331422 | .1742902 |
| Age 25-34 | .3251689 | .0136195 | .2984479 | .3518899 |
| Age 35-44 | .2516892 | .0126177 | .2269336 | .2764448 |
| Age 45-54 | .1613176 | .0106942 | .1403359 | .1822992 |
| Age 55-64 | .0625 | .0070377 | .0486922 | .0763078 |
|  |  |  |  |  |
| Marital status |  |  |  |  |
| Married | .6875 | .0134762 | .66106 | .71394 |
| Not Married | .2119932 | .0118832 | .1886787 | .2353078 |
| Others | .1005068 .0087419 .0833555 .117658 | | | |

**Table 6-b : Mean Jouf**

|  | | | | |
| --- | --- | --- | --- | --- |
| Mean estimation Number of obs = 193 | | | | |
|  | | | | |
|  | | | | |
|  | Mean | Std. Err. | [95% Conf. Interval] | |
|  |  |  |  | |
| Male | .5336788 | .0360024 | | .4626677 .6046898 |
|  |  |  | |  |
| Education |  |  | |  |
| High school | .6373057 | .0346971 | | .5688692 .7057422 |
| University and above | .3626943 | .0346971 | | .2942578 .4311308 |
|  |  |  | |  |
| Smoker | .2642487 | .0318216 | | .201484 .3270134 |
|  |  |  | |  |
| Income (Saudi Riyals) |  |  | |  |
| Less than 3,000 | .2020725 | .0289791 | | .1449143 .2592308 |
| 3,000 to 5,000 | .1865285 | .0281121 | | .1310803 .2419767 |
| 5,000 to 7,000 | .2901554 | .0327526 | | .2255542 .3547566 |
| 7,000 to 10,000 | .3212435 | .0336995 | | .2547747 .3877123 |
|  |  |  | |  |
| Age 15-24 | .3108808 | .0334036 | | .2449956 .376766 |
| Age 25-34 | .388601 | .0351774 | | .3192173 .4579848 |
| Age 35-44 | .2849741 | .0325771 | | .2207191 .3492291 |
| Age 45-54 | 0 (omitted) |  | |  |
| Age 55-64 | 0 (omitted) |  | |  |
|  |  |  | |  |
| Marital status |  |  | |  |
| Married | .6632124 | .0341078 | | .5959383 .7304865 |
| Not Married | .2953368 | .032923 | | .2303996 .3602739 |

**Table 6-c : Mean Jazan**

| Mean estimation Number of obs = 616 | | | | |
| --- | --- | --- | --- | --- |
|  | | | | |
|  | | | | |
|  | | Mean | Std. Err. | [95% Conf. Interval] |
|  | |  |  |  |
| Male | | .4104235 | . 0198681 | .3714057 .4494412 |
|  | |  |  |  |
| Education | |  |  |  |
| Illiterate | | .3355049 | .0190706 | .2980532 .3729566 |
| High School | | .4641694 | .0201429 | .4246119 .5037268 |
| University and above | | .2003257 | .0161657 | .1685788 .2320726 |
|  | |  |  |  |
| Smoker | | .1302932 | .0135962 | .1035924 .1569939 |
|  | |  |  |  |
| Income (Saudi Riyals) | |  |  |  |
| Less than 3,000 | | .2605863 | .0177292 | .225769 .2954037 |
| 3,000 to 5,000 | | .1547231 | .0146065 | .1260382 .183408 |
| 5,000 to 7,000 | | .1482085 | .0143507 | .120026 .176391 |
| 7,000 to 10,000 | | .1807818 | .0155434 | .1502569 .2113066 |
| 10,000 to 15,000 | | .1514658 | .0144798 | .1230298 .1799018 |
| 15,000 to 20,000 | | .0618893 | .0097321 | .042777 .0810015 |
| 20,000 to 30,000 | | .0423453 | .0081335 | .0263724 .0583182 |
|  | |  |  |  |
| Age 15-24 | | .2394137 | .0172353 | .2055663 .2732611 |
| Age 25-34 | | .2263844 | .0169027 | .1931902 .2595786 |
| Age 35-44 | | .1938111 | .0159653 | .1624577 .2251644 |
| Age 45-54 | | .1058632 | .0124264 | .0814598 .1302666 |
| Age 55-64 | | .1026059 | .012256 | .0785371 .1266746 |
|  | |  |  |  |
| Marital status | |  |  |  |
| Married | .5977199 | | .0198054 | .5588253 .6366145 |

**Table 6-d: Mean Baha**

|  | | | | | |
| --- | --- | --- | --- | --- | --- |
| Mean estimation | **Number of obs = 514** | | | | |
|  |  | | | | |
|  |  | | | | |
|  | Mean | | Std. Err. | [95% Conf. Interval] | |
|  |  | |  |  | |
| Male | .4747082 | | .0220473 | .4313941 | .5180222 |
|  |  | |  |  |  |
| Education |  | |  |  |  |
| Illiterate | .3054475 | | .0203358 | .2654957 | .3453992 |
| High School | .4357977 | | .0218928 | .3927871 | .4788082 |
| University and above | .2587549 | | .019336 | .2207674 | .2967423 |
|  |  | |  |  |  |
| Smoker | .1848249 | | .0171375 | .1511566 | .2184932 |
|  |  | |  |  |  |
| Income (Saudi Riyals) |  | |  |  |  |
| Less than 3,000 | .1964981 | | .0175434 | .1620323 | .2309638 |
| 3,000 to 5,000 | .1439689 | | .0154996 | .1135184 | .1744194 |
| 5,000 to 7,000 | .1245136 | | .0145772 | .0958752 | .153152 |
| 7,000 to 10,000 | .1770428 | | .0168527 | .143934 | .2101516 |
| 10,000 to 15,000 | .2237354 | | .0183998 | .1875871 | .2598837 |
| 15,000 to 20,000 | .1050584 | | .013538 | .0784617 | .1316551 |
| 20,000 to 30,000 | .0175097 | | .0057909 | .006133 | .0288865 |
|  |  | |  |  |  |
| Age 15-24 | .2042802 | | .0178006 | .1693091 | .2392512 |
| Age 25-34 | .2081712 | | .0179253 | .1729551 | .2433873 |
| Age 35-44 | .2062257 | | .0178633 | .1711315 | .2413199 |
| Age 45-54 | .1245136 | | .0145772 | .0958752 | .153152 |
| Age 55-64 | .1070039 | .0136479 | | .0801912 | .1338165 |
|  |  |  | |  |  |
| Marital status |  | |  |  |  |
| Married | .6342412 | | .021265 | .592464 | .6760185 |
| Not Married | .233463 | | .0186774 | .1967694 | .2701567 |
| Divorced, Separated and Widowed | .1322957 | | .0149589 | .1029075 | .161684 |

**Table 7: Differences between the full sample and the usable sample with respect to Education and Gender**

**Table 7-a: Mean for Used Sample [Education]**

| Mean estimation Number of obs = 7,603 | | | | |
| --- | --- | --- | --- | --- |
|  | Mean | Std. Err. | [95% Conf.  Interval] | |
|  |  |  |  |  |
| illiterate | 0.2093 | 0.0047 | 0.2001 | 0.2184 |
| High School | 0.4980 | 0.0057 | 0.4867 | 0.5092 |
| degree and above | 0.2928 | 0.0052 | 0.2825 | 0.3030 |

**Table 7-b: Mean for Full Sample [Education]**

| Mean estimation Number of obs = 9,574 | | | | |
| --- | --- | --- | --- | --- |
|  | Mean | Std. Err. | [95% Conf. Interval] | |
|  |  |  |  |  |
| illiterate | 0.2291 | 0.0043 | 0.2206 | 0.2375 |
| High School | 0.5025 | 0.0051 | 0.4925 | 0.5125 |
| degree and above | 0.2684 | 0.0045 | 0.2596 | 0.2773 |

**Table 7-c: Mean for** excluded **Sample [Education]**

| Mean estimation Number of obs = 1,971 | | | | |
| --- | --- | --- | --- | --- |
|  | Mean | Std. Err. | [95% Conf. Interval] | |
|  |  |  |  |  |
| illiterate | 0.3054 | 0.0104 | 0.2851 | 0.3258 |
| High School | 0.5200 | 0.0113 | 0.4980 | 0.5421 |
| degree and above | 0.1745307 | 0.0085517 | 0.1577593 | 0.1913021 |

**Table 7-d : Mean for Used Sample [Gender]**

| Mean estimation Number of obs = 7,603 | | | | |
| --- | --- | --- | --- | --- |
|  | Mean | Std. Err. | [95% Conf. Interval] | |
| Gender |  |  |  |  |
| Female | 0.4848 | 0.0057 | 0.4736 | 0.4960 |
| Male | 0.5152 | 0.0057 | 0.5040 | 0.5264 |

**Table 7-e: Mean for full Sample [Gender]**

| Mean for full Sample [Gender] | | | | |
| --- | --- | --- | --- | --- |
| Mean estimation Number of obs = 10,821 | | | | |
|  | Mean | Std. Err. | [95% Conf. Interval] | |
| Gender |  |  |  |  |
| Female | 0.5106 | 0.0048 | 0.5012 | 0.5200 |
| Male | 0.4894 | 0.0048 | 0.4800 | 0.4988 |

**Table 7-f: Mean for excluded Sample [Gender]**

| Mean estimation Number of obs = 3,218 | | | | |
| --- | --- | --- | --- | --- |
|  |  |  |  |  |
|  | Mean | Std. Err. | [95% Conf. Interval] | |
| Gender |  |  |  |  |
| Female | 0.571473 | 0.0087249 | 0.554366 | 0.5885799 |
| Male | 0.428527 | 0.0087249 | 0.4114201 | 0.445634 |

Appendix 2:

The salary ranges in Riyals, when converted to Euros, are approximately as follows:

Less than 3,000 Riyals per month → 0 to 750 Euros per month

3,000 to 5,000 Riyals per month → 750 to 1,250 Euros per month

5,000 to 7,000 Riyals per month → 1,250 to 1,750 Euros per month

7,000 to 10,000 Riyals per month → 1,750 to 2,500 Euros per month

10,000 to 15,000 Riyals per month → 2,500 to 3,750 Euros per month

15,000 to 20,000 Riyals per month → 3,750 to 5,000 Euros per month

20,000 to 30,000 Riyals per month → 5,000 to 7,500 Euros per month

30,000 Riyals or more per month → 7,500 Euros or more per month
